# The poor performance of cardiovascular risk scores in identifying patients with idiopathic inflammatory myopathies at high cardiovascular risk

**DOI:** 10.1515/med-2023-0703

**Published:** 2023-05-17

**Authors:** Li Qin, Qiang Luo, Yinlan Hu, Shuangshuang Yan, Xiaoqian Yang, Yiwen Zhang, Feng Xiong, Han Wang

**Affiliations:** Department of Cardiology, The Affiliated Hospital of Southwest Jiaotong University, The Third People’s Hospital of Chengdu, Chengdu, 610031, Sichuan, China; Department of Cardiology, The Third Xiangya Hospital, Central South University, Changsha, 410013, China; Department of Cardiology, The Affiliated Hospital of Southwest Jiaotong University, The Third People’s Hospital of Chengdu, No. 82, Qinglong Street,, Chengdu, 610031, Sichuan, China

**Keywords:** autoimmune diseases, cardiovascular risk score, subclinical atherosclerosis

## Abstract

Framingham risk score (FRS), systematic coronary risk evaluation (SCORE), the 10-year atherosclerotic cardiovascular disease risk algorithm (ASCVD), and their modified risk scores are the most common cardiovascular risk scores. The aim of this case–control study was to evaluate the performance of cardiovascular risk scores in detecting carotid subclinical atherosclerosis (SCA) in patients with idiopathic inflammatory myopathies (IIMs). A total of 123 IIMs patients (71.5% female, mean age 50 ± 14 years) and 123 age- and gender-matched healthy controls were included in this study. Carotid SCA was more prevalent in IIMs patients compared with controls (77.2 vs 50.4%, *P* < 0.001). Moreover, patients with carotid SCA+ had older age, and all risk scores were significantly higher in IIMs patients with SCA+ compared to subjects with SCA− (all *P* < 0.001). According to FRS, SCORE, and ASCVD risk scores, 77.9, 96.8, and 66.7% patients with SCA+ were classified as low risk category, respectively. The modified scores also demonstrated a modest improvement in sensitivity. Notably, by adopting the optimal cutoff values, these risk scores had good discrimination on patients with SCA+, with area under curves of 0.802–0.893. In conclusion, all cardiovascular risk scores had a poor performance in identifying IIMs patients at high cardiovascular risk.

## 1 Introduction

Idiopathic inflammatory myopathies (IIMs) are a group of relatively rare systemic autoimmune diseases, and the annual prevalence of IIMs ranges from 14.0 to 17.4 per 100,000 person-years [1,2]. The heart is an important target organ in IIMs patients, but cardiac involvement typically remains silent that seldom attracts the attention of clinicians [3]. However, numerous studies demonstrated that atherosclerotic cardiovascular diseases (ASCVD) such as myocardial infarction and stroke were the leading cause of death in IIMs patients, and the risk of cardiovascular diseases (CVD) in these patients was increased in comparison with the general population [3–5]. Therefore, early and accurate identification of CVD risk is essential to reduce the incidence of cardiovascular events and improve the prognosis of IIMs patients. At present, the assessment of cardiovascular risk can be based on risk assessment algorithms or imaging, specifically on vascular ultrasound. In the general population, Framingham risk score (FRS), systematic coronary risk evaluation (SCORE), and the 10-year ASCVD risk algorithm are the most commonly used risk scores [6–8]. Nevertheless, the majority of studies revealed that the above risk scores cannot accurately reflect the actual cardiovascular risk of patients with autoimmune diseases, such as systemic lupus erythematosus (SLE), rheumatoid arthritis (RA), and psoriatic arthritis (PsA) [9–11]. Furthermore, the performance of EULAR-modified risk score (a 1.5 multiplication factor for risk score) in RA patients was still unsatisfactory [12,13].

Recent studies have confirmed that subclinical atherosclerosis (SCA) is a reliable surrogate marker of CVD, and several non-invasive imaging techniques can be employed to determine SCA [14,15]. Among them, carotid ultrasonography is a simple, rapid, sensitive, reproducible, and relatively cheap tool for identifying and quantifying SCA, which can detect increased carotid intimal-media thickness (IMT) and the presence of plaque [16,17]. To date, the reports on SCA in patients with IIMs are relatively rare. Limited evidence indicated that patients with IIMs had increased carotid IMT compared to healthy controls, implying that IIMs patients may have a higher risk of SCA than the general population [18,19]. Moreover, only one study on patients with antisynthetase syndrome (a special subtype of IIMs) points out that SCORE/mSCORE has a poor performance in assessing patients at high cardiovascular risk, but no detailed study has been conducted to test the performance of other risk scores in IIMs patients [19]. Thus, the primary aim of the current study is to identify the feasibility of FRS, SCORE, and ASCVD in IIMs patients at high cardiovascular risk. Besides, it also explores potential factors associated with carotid SCA in patients with IIMs.

## 2 Methods

### 2.1 Study population

This case–control study was conducted in the Third People’s Hospital of Chengdu. Cases were identified from the individuals who were diagnosed with IIMs referred to the Cardiology and Rheumatology Department between January 2016 and January 2021. Controls were randomly selected from healthy individuals who received physical examination at the same period and were matched to cases based on gender and age (within 3 years). All IIMs patients were diagnosed in accordance with the criteria of Bohan and Peter by skilled clinicians [20]. Patients were excluded if they had previous history of malignant tumor, cardiovascular events (ischemic heart disease, cerebrovascular disease, peripheral arterial disease, or heart failure), respiratory failure, renal failure, severe liver impairment, severe infectious diseases, as well as other connective tissue diseases, such as SLE, RA, and mixed connective tissue disease. In addition, elderly patients (age >80 years) and those <20 years old, and subjects with incomplete data were also not included. The same exclusion criteria were also applied to the control group. Finally, a total of 123 consecutive IIMs patients and 123 healthy subjects were included in this study, and they all underwent carotid ultrasonography. The current study was ethical approved by the ethics committee of the Third People’s Hospital of Chengdu (2019-S-20), and written informed consent was taken from each participant according to the Declaration of Helsinki.

### 2.2 Data collection and definition of variables

A self-designed questionnaire drawn up by the research team was used to collect the information of participants. Demographics, clinical manifestations, laboratory data, and treatments were systematically extracted from electronic medical records by two trained medical students and reviewed by a senior clinician. Venous blood samples were collected from all individuals in the morning (after at least 12 h of fasting) for routine laboratory analysis by following standard procedures. The following laboratory parameters were measured: fasting blood glucose (FBG), serum urea, serum creatinine (Scr), triglyceride (TG), total cholesterol (TC), high-density lipoprotein-cholesterol (HDL-C), low-density lipoprotein-cholesterol (LDL-C), C-relative protein (CRP), erythrocyte sedimentation rate (ESR), antinuclear antibodies (ANA), anti SSA antibody, anti SSB antibody, anti-Jo1 antibody, and creatine kinase (CK). The values of ESR ≥40 mm/h and CRP ≥10 mg/L were considered positive. Drug administration referred to the use of antihypertensive drugs, hypoglycemic drugs, glucocorticoids (GC), and disease modifying anti-rheumatic drugs (DMARDs) within 1 month before admission. In addition, lung involvement of IIMs patients was evaluated by high resolution CT, which mainly included pulmonary infection, pulmonary interstitial fibrosis, and pleural effusion.

The diagnostic criteria of hypertension were systolic blood pressure (SBP) ≥140 mmHg and/or diastolic blood pressure (DBP) ≥90 mmHg, or use of antihypertensive drugs [21]. Diabetes mellitus was defined as FBG ≥7.0 mmol/L and/or a plasma glucose level ≥11.1 mmol/L at 2 h after a 75 g glucose load, and/or use of oral hypoglycemic agents or insulin [22]. Smokers were defined as individuals who were smoking at least one cigarette per day for more than 1 year or had recently stopped smoking within the last year, and the rest of subjects were considered as non-smokers [23].

### 2.3 Cardiovascular risk assessment

Cardiovascular risk assessment was performed using clinical risk scores and ultrasound imaging of carotid arteries. In the present study, online software was used for calculating FRS, SCORE, and ASCVD risk score in IIMs patients. In addition, EULAR-modified scores (multiplied by 1.5) were calculated for all risk scores and labeled with the prefix “m-.” Patients with FRS/m-FRS risk >10% [7], SCORE/m-SOCRE risk score >5% [6], and ASCVD/m-ASCVD risk score >7.5% [8] were considered as having high risk of CVD. Carotid IMT was measured by high-resolution B-mode ultrasonography using a Philips iE 33 machine equipped with a 7–13 MHz linear vascular probe (Philips Healthcare, USA) [9]. As previously described, carotid IMT was measured at six locations, including the left and right distal common carotid arteries (10 mm proximal to the carotid bulb), carotid bulbs, and proximal internal carotid arteries (10 mm distal to the carotid bifurcation) [11]. The average IMT of six carotid segments was used for further analysis. Carotid plaque presence was defined as a focal wall thickness (IMT ≥1.5 mm), or increased by at least 0.5 mm or 50% compared with the IMT of the adjacent vascular wall [24]. Patients with average IMT ≥0.9 mm and/or the presence of plaque were considered as SCA+ [25]. It was worth noting that the carotid IMT of all participants was measured by a highly skilled sonographer and was read by an experienced cardiologist. Moreover, they were blinded to patients’ clinical information.

### 2.4 Statistical analysis

Categorical variables were expressed as frequency (percentage), and quantitative variables were described as mean ± standard deviation or median (interquartile range) according to data distribution. Comparisons between both groups were performed with the independent sample *t*-test or Mann–Whitney *U*-test for continuous variables, and Chi-square test or Fisher’s exact test for categorical variables. Binary logistic regression analysis was used to identify the related factors of SCA in patients with IIMs. Notably, before the binary logistic regression analysis, multi-collinearity was carried out on all the statistically significant variables using the variance inflation factor (VIF). In general, there was no multi-collinearity among the variables when all values of VIF were less than 10. To evaluate the diagnostic performance of cardiovascular risk scores in comparison to carotid ultrasonography, the receiver operating characteristic (ROC) curve analysis was performed. The optimal cutoff values of the cardiovascular risk scores were obtained according to the sensitivity and specificity at the point where the Youden index was maximized. The Hosmer–Lemeshow test was applied to assess the goodness of fit for the observed and expected risk of carotid SCA+ estimated by the cardiovascular risk scores. The larger the *P*-value of Hosmer–Lemeshow test, the better the fit. All statistical analyses were conducted using IBM SPSS Version 26 (SPSS, Chicago, USA). All tests were two-tailed, and *P*-value <0.05 was considered statistically significant.

## 3 Results

### 3.1 Subjects’ characteristics and cardiovascular risk scores

A total of 123 patients with IIMs (71.5% female, mean age 50 ± 14 years) and 123 age- and gender-matched healthy subjects were included in this study. The detailed characteristics between cases and controls are shown in Table 1. The case group consisted of 36 patients with polymyositis, 83 patients with dermatomyositis, and 4 patients with inclusion body myositis, with the median disease duration of 6 months. In addition, IIMs patients had higher levels of TG (2.0 vs 1.1 mmol/L), LDL-C (3.5 vs 2.7 mmol/L), and serum urea (5.5 vs 5.0 mmol/L), lower levels of FBG (4.6 vs 4.9 mmol/L) and Scr (51.8 vs 63.0 μmol/L) compared to controls. Carotid ultrasound revealed that 77.2% of IIMs patients and 50.4% healthy controls had carotid SCA+, and the difference between the two groups was statistically significant (*P* < 0.001). Different cardiovascular risk scores had different applicable conditions, especially ASCVD risk score was applied to the study population aged 40–79 years. In the current study, there were 30 subjects younger than 40 years old in both the case and control groups. Finally, the data of 123, 123, and 93 individuals were qualified to calculate FRS/m-FRS, SCORE/m-SCORE, and ASCVD/m-ASCVD risk score in both the groups, respectively. The results demonstrated that all cardiovascular risk scores were not significantly different between IIMs patients and controls.

**Table 1:** Main characteristics of IIMs patients and controls

Vaiables	IIMs (*n* = 123)	Controls (*n* = 123)	*P*-value
Age (years)	50 ± 14	50 ± 14	0.731
Gender (*n*, %)			1.000
Female	88 (71.5)	88 (71.5)	
Male	35 (28.5)	35 (28.5)	
Smoking (*n*, %)	8 (6.5)	13 (10.6)	0.254
Hypertension (*n*, %)	26 (21.1)	18 (14.6)	0.183
Diabetes mellitus (*n*, %)	16 (13.0)	14 (11.4)	0.697
SBP (mmHg)	122 ± 18	119 ± 15	0.090
DBP (mmHg)	75 (65–84)	74 (68–79)	0.231
FBG (mmol/L)	4.6 (4.2–5.5)	4.9 (4.6–5.6)	0.001
TG (mmol/L)	2.0 (1.6–2.6)	1.1 (0.9–1.5)	<0.001
TC (mmol/L)	5.7 ± 1.1	4.7 ± 1.1	0.995
HDL-C (mmol/L)	1.5 (1.1–1.7)	1.3 (1.2–1.6)	0.231
LDL-C (mmol/L)	3.5 (3.1–3.8)	2.7 (2.2–3.3)	<0.001
Serum urea (mmol/L)	5.5 (4.3–7.0)	5.0 (4.1–5.9)	0.003
Serum creatinine (μmol/L)	51.8 (41.2–61.4)	63.0 (57.9–71.4)	<0.001
Use of antihypertensive drugs (*n*, %)	4 (3.3)	6 (6.9)	0.518
SCA+ (*n*, %)	95 (77.2)	62 (50.4)	<0.001
FRS (%)	2 (0.5–6)	1 (0.5–5)	0.062
mFRS (%)	3 (0.8–9)	1.5 (0.8–7.5)	0.062
SCORE (%)	0.4 (0.1–1.4)	0.4 (0.1–1.1)	0.907
mSCORE (%)	0.6 (0.2–2.1)	0.6 (0.2–1.5)	0.907
ASCVD (%)	3.2 (1.7–11.4)	2.9 (1.2–6.1)	0.226
mASCVD (%)	4.8 (2.6–17.1)	4.4 (1.8–9.2)	0.226
Disease duration (*n*, %) (months)			—
<6	49 (39.8)	—	
≥6	74 (60.2)	—	
Dysphagia (*n*, %)	32 (26.0)	—	—
Myalgia (*n*, %)	65 (52.8)	—	—
Arthralgia (*n*, %)	40 (32.5)	—	—
Rash (*n*, %)	84 (68.3)	—	—
Lung involvement (*n*, %)	58 (47.2)	—	—
Gottron’s sign (*n*, %)	26 (21.1)	—	—
Raynaud’s phenomenon (*n*, %)	12 (9.8)	—	—
ESR positive (*n*, %)	41 (33.3)	—	—
CRP positive (*n*, %)	52 (42.3)	—	—
ANA positive (*n*, %)	79 (64.2)	—	—
Anti SSA antibody positive (*n*, %)	9 (7.3)	—	—
Anti SSB antibody positive (*n*, %)	5 (4.1)	—	—
Anti-Jo1 antibody positive (*n*, %)	6 (4.9)	—	—
CK (IU/L)	198 (54–1,762)	—	—
Use of GC (*n*, %)	60 (48.8)	—	—
Use of MTX (*n*, %)	19 (15.4)	—	—
Use of CTX (*n*, %)	3 (2.4)	—	—
Use of HCQ (*n*, %)	10 (8.1)	—	—
Use of AZA (*n*, %)	5 (4.1)	—	—
Use of TII (*n*, %)	7 (5.7)	—	—
Use of TGP (*n*, %)	4 (3.3)	—	—

Abbreviations: IIMs, idiopathic inflammatory myopathies; SBP, systolic blood pressure; DBP, diastolic blood pressure; FBG, fasting blood glucose; TG, triglyceride; TC, total cholesterol; HDL-C, high-density lipoprotein-cholesterol; LDL-C, low-density lipoprotein-cholesterol; SCA, subclinical atherosclerosis; FRS, Framingham risk score; SCORE, systematic coronary risk evaluation; ASCVD, atherosclerotic cardiovascular disease risk algorithm; ESR, erythrocyte sedimentation rate; CRP, C-relative protein; ANA, antinuclear antibodies; CK, creatine kinase; GC, glucocorticoid; MTX, methotrexate; CTX, cyclophosphamide; HCQ, hydroxychloroquine; AZA, azathioprine; TII, *Tripterygium wilfordii*; TGP, total glucosides of paeony.

### 3.2 Performance of cardiovascular risk scores in IIMs patients

**Figure 1:**
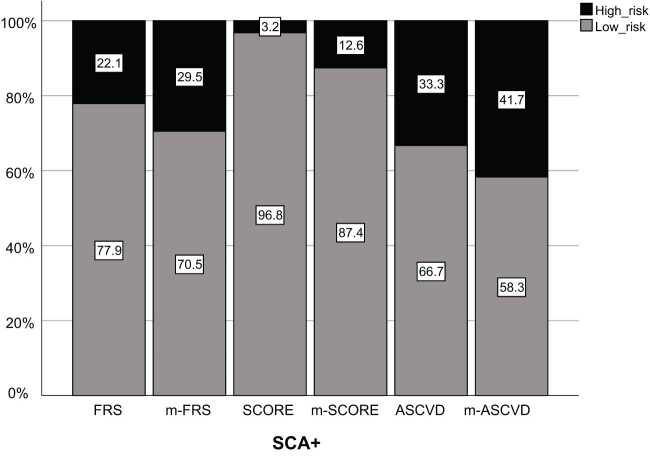
Cardiovascular risk scores and EULAR-modified risk scores in discriminating carotid subclinical atherosclerosis using the preset cutoff values. Abbreviations: FRS, Framingham risk score; SCORE, systematic coronary risk evaluation; ASCVD, atherosclerotic cardiovascular disease risk algorithm.

**Figure 2:**
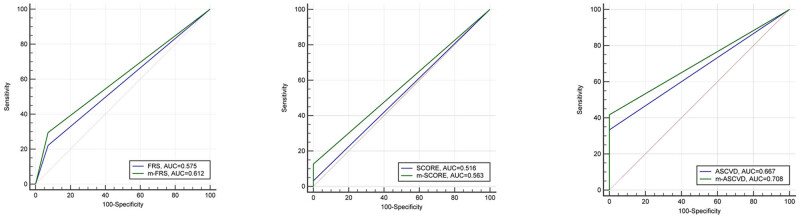
ROC curve of the risk scores and modified risk scores in discriminating subclinical atherosclerosis. Abbreviations: FRS, Framingham risk score; SCORE, systematic coronary risk evaluation; ASCVD, atherosclerotic cardiovascular disease risk algorithm; AUC, area under the curve.

There were 95 IIMs patients who were defined as carotid SCA+ with average IMT ≥0.9 mm and/or the presence of carotid plaque. As presented in Table 2, all cardiovascular risk scores were significantly higher in patients with SCA+ compared with those with SCA− (FRS: 2 vs 0.5%; SCORE: 0.6 vs 0.07%; ASCVD: 3.7 vs 1.0%; all *P* < 0.001). However, by adopting the preset cutoff values, only 21 (22.1%), 3 (3.2%), and 28 (33.3%) patients with SCA+ were classified as high risk category according to FRS, SCORE, and ASCVD, respectively. Considering that systemic autoimmune diseases often share many clinical and laboratory features, we also tested whether modified risk scores would enhance the diagnostic accuracy in IIMs patients. The findings found that EULAR modified scores increased the sensitivity of m-FRS, m-SCORE, and m-ASCVD in discriminating against carotid SCA from 22.1 to 29.5%, 3.2 to 12.6%, and 33.3 to 41.7%, respectively (Figure 1). It was worth mentioning that 7.1% patients with SCA− were also categorized as high risk by applying FRS and m-FRS. Nevertheless, none of the patients with SCA− was included in the high risk group by applying SCORE, m-SCORE, ASCVD, and m-ASCVD. ROC analysis was conducted to evaluate the performance of cardiovascular risk scores in discriminating patients with high risk, and the results were not satisfactory. The areas of the ROC curve were 0.575 (95% CI: 0.461–0.688) for FRS, 0.612 (95% CI: 0.503–0.721) for m-FRS, 0.516 (95% CI: 0.396–0.636) for SCORE, 0.563 (95% CI: 0.450–0.677) for m-SCORE, 0.677 (95% CI: 0.521–0.812) for ASCVD, and 0.708 (95% CI: 0.576–0.840) for m-ASCVD (Figure 2). From the above results, we found that the performance of ASCVD/m-ASCVD was better than FRS/m-FRS and SCORE/m-SCORE, but the former performed suboptimal in identifying the actual high CVD risk in IIMs patients with carotid SCA.

**Table 2:** Characteristics of IIMs patients with SCA+ and SCA−

Vaiables	SCA+ (*n* = 95)	SCA− (*n* = 28)	*P*-value
Age (years)	55 ± 12	35 ± 9	<0.001
Gender (*n*, %)			0.623
Female	69 (72.6)	19 (67.9)	
Male	26 (27.4)	9 (32.1)	
Smoking (*n*, %)	7 (7.4)	1 (3.6)	0.779
Hypertension (*n*, %)	25 (26.3)	1 (3.6)	0.010
Diabetes mellitus (*n*, %)	14 (14.7)	2 (7.1)	0.465
Disease duration (*n*, %) (months)			0.418
<6	36 (37.9)	13 (46.4)	
≥6	59 (62.1)	15 (53.6)	
SBP (mmHg)	125 ± 17	109 ± 14	<0.001
DBP (mmHg)	78 (68–85)	67 (59–80)	0.002
FBG (mmol/L)	4.6 (4.2–5.7)	4.6 (4.1–5.2)	0.679
TG (mmol/L)	1.9 (1.5–2.6)	2.0 (1.6–2.6)	0.798
TC (mmol/L)	5.8 ± 1.0	5.5 ± 1.4	0.314
HDL-C (mmol/L)	1.4 (1.1–1.7)	1.5 (1.1–1.7)	0.988
LDL-C (mmol/L)	3.5 (3.1–3.9)	3.5 (2.9–3.7)	0.606
Serum urea (mmol/L)	5.6 (4.5–7.5)	5.2 (4.1–6.0)	0.031
Serum creatinine (μmol/L)	54.8 ± 17.0	45.3 ± 14.0	0.008
Dysphagia (*n*, %)	26 (27.4)	6 (21.4)	0.529
Myalgia (*n*, %)	50 (52.6)	15 (53.6)	0.930
Arthralgia (*n*, %)	28 (29.5)	12 (42.9)	0.184
Rash (*n*, %)	66 (69.5)	18 (64.3)	0.604
Lung involvement (*n*, %)	47 (49.5)	11 (39.3)	0.343
Gottron’s sign (*n*, %)	19 (20.0)	7 (25.0)	0.569
Raynaud’s phenomenon (*n*, %)	9 (9.5)	3 (10.7)	1.000
ESR positive (*n*, %)	29 (30.5)	12 (42.9)	0.224
CRP positive (*n*, %)	38 (40.0)	14 (50.0)	0.347
ANA positive (*n*, %)	64 (67.4)	15 (53.6)	0.181
Anti SSA antibody positive (*n*, %)	8 (8.4)	1 (3.6)	0.650
Anti SSB antibody positive (*n*, %)	4 (4.2)	1 (3.6)	1.000
Anti-Jo1 antibody positive (*n*, %)	4 (4.2)	2 (7.2)	0.893
CK (IU/L)	153 (53–1,394)	287 (55–4,948)	0.538
Use of antihypertensive drugs (*n*, %)	4 (4.2)	0 (0.0)	0.573
Use of GC (*n*, %)	45 (47.4)	15 (53.6)	0.564
Use of MTX (*n*, %)	14 (14.7)	5 (17.9)	0.917
Use of CTX (*n*, %)	3 (3.2)	0 (0.0)	1.000
Use of HCQ (*n*, %)	7 (7.4)	3 (10.7)	0.860
Use of AZA (*n*, %)	4 (4.2)	1 (3.6)	1.000
Use of TII (*n*, %)	6 (6.3)	1 (3.6)	0.931
Use of TGP (*n*, %)	3 (3.2)	1 (3.6)	1.000
FRS (%)	2 (1–8)	0.5 (0.5–1)	<0.001
mFRS (%)	3 (1.5–12)	0.8 (0.8–1.5)	<0.001
SCORE (%)	0.6 (0.22–2.28)	0.07 (0.03–0.13)	<0.001
mSCORE (%)	0.9 (0.33–3.42)	0.1 (0.05–0.20)	<0.001
ASCVD (%)	3.7 (1.9–12.4)	1.0 (0.6–1.8)	<0.001
mASCVD (%)	5.6 (2.9–18.6)	1.5 (0.9–2.7)	<0.001

Abbreviations: IIMs, idiopathic inflammatory myopathies; SCA, subclinical atherosclerosis; SBP, systolic blood pressure; DBP, diastolic blood pressure; FBG, fasting blood glucose; TG, triglyceride; TC, total cholesterol; HDL-C, high-density lipoprotein-cholesterol; LDL-C, low-density lipoprotein-cholesterol; ESR, erythrocyte sedimentation rate; CRP, C-relative protein; ANA, antinuclear antibodies; CK, creatine kinase; GC, glucocorticoid; MTX, methotrexate; CTX, cyclophosphamide; HCQ, hydroxychloroquine; AZA, azathioprine; TII, *Tripterygium wilfordii*; TGP, total glucosides of paeony; FRS, Framingham risk score; SCORE, systematic coronary risk evaluation; ASCVD, atherosclerotic cardiovascular disease risk algorithm.

Notably, by adopting the optimal cutoff values (FRS >1.5%, SCORE >0.14%, and ASCVD >1.5%), the performance of these risk scores were in good agreement with carotid SCA, and the areas of the ROC curve were 0.802 (95% CI: 0.721–0.868) for FRS, 0.893 (95% CI: 0.824–0.941) for SCORE, and 0.860 (95% CI: 0.773–0.924) for ASCVD. Besides, the *P*-value of Hosmer–Lemeshow test was 0.085 for FRS, 0.185 for SCORE, and 0.239 for ASCVD, which indicated that these cardiovascular risk scores had a moderate fit. Therefore, we assumed that the lower high-risk threshold may improve the diagnostic performance between cardiovascular risk scores and carotid SCA. Sensitivity and specificity of the different cutoff values are presented in Table 3.

**Table 3:** Sensitivity and specificity of preset and modified cutoffs for risk scores, and cutoffs with highest overall accuracy

Risk scores	Cutoff (%)	Sensitivity (%)	Specificity (%)	AUC
Highest Youden index				
FRS	1.5	66.3	85.7	0.802
SCORE	0.14	88.4	78.6	0.893
ASCVD	1.5	82.1	77.8	0.860
Preset				
FRS	10	22.1	92.9	0.575
SCORE	5	3.2	100	0.516
ASCVD	7.5	33.3	100	0.667
Modified				
m-FRS	10	29.5	92.9	0.612
m-SCORE	5	12.6	100	0.563
m-ASCVD	7.5	41.7	100	0.708

Abbreviations: AUC, area under curve; FRS, Framingham risk score; SCORE, systematic coronary risk evaluation; ASCVD, atherosclerotic cardiovascular disease risk algorithm.

### 3.3 Predictors of carotid SCA in IIMs patients

The demographics, clinical manifestations, laboratory data, and treatments of SCA+ and SCA− patients are summarized in Table 2. The patients with SCA+ were significantly older (55 ± 12 vs 35 ± 9 years, *P* < 0.001), had higher levels of SBP (125 ± 17 vs 109 ± 14 mmHg, *P* < 0.001), DBP (78 vs 67 mmHg, *P* = 0.002), serum urea (5.6 vs 5.2 mmol/L, *P* = 0.031), and Scr (54.8 ± 17.0 vs 45.3 ± 14.0 mmol/L, *P* = 0.008). In addition, hypertension was indeed more prevalent among patients with SCA+ compared with individuals with SCA− (26.3 vs 3.6%, *P* = 0.01). The numbers of smoking and diabetes mellitus were higher in patients with SCA+, but there was no statistically significant difference between the two groups (all *P* > 0.05). The collinearity diagnostics indicated that there was no obvious multi-collinearity among variables, with the value of VIF ranging from 1.244 to 3.501. All variables which showed statistically significant in univariate analyses were included in the binary logistic regression model, and the results presented that age (OR = 1.160, 95%CI: 1.083–1.242, *P* < 0.001) was associated with carotid SCA in IIMs patients (Table 4).

**Table 4:** Associated factors of subclinical atherosclerosis in IIMs patients

Variables	*β*	OR (95% CI)	*P*-value
Age (years)	0.148	1.160 (1.083–1.242)	<0.001
SBP (mmHg)	0.075	1.078 (0.997–1.167)	0.061
DBP (mmHg)	−0.059	0.943 (0.852–1.042)	0.249
Hypertension	0.582	1.789(0.164–19.567)	0.634
Serum urea (mmol/L)	0.157	1.170 (0.801–1.708)	0.417
Serum creatinine (μmol/L)	0.003	1.003 (0.959–1.048)	0.908

Abbreviations: IIMs, idiopathic inflammatory myopathies; SBP, systolic blood pressure; DBP, diastolic blood pressure.

## 4 Discussion

In the current study, IIMs patients showed a frequent carotid SCA+ compared with the general population, and all cardiovascular risk scores were higher in IIMs patients with SCA+ than in cases with SCA− (all *P* < 0.001). By adopting the optimal cutoff values, these risk scores had good discrimination in carotid SCA, with area under the ROC curves of 0.802–0.893. The Hosmer–Lemeshow test suggested that these cardiovascular risk scores had moderate goodness of fit (*P* ranging from 0.085 to 0.239). However, by adopting the preset cutoff values, there was a poor agreement between these risk scores and carotid SCA, with area under the ROC curves of 0.516–0.667. When the 1.5 multiplication factor was introduced to the risk scores, the sensitivity of these risk scores in discriminating against carotid SCA only had a slight increase.

More recently, some studies confirmed that there was a relatively poor consistency between the above cardiovascular risk scores and carotid ultrasonography in patients with autoimmune diseases, such as SLE, PsA, as well as antisynthetase syndrome, which was consistent with the results of this study [9,19,26]. In patients with IIMs, the increased risk of CVD was not only attributed to traditional risk factors, such as age, hypertension, dyslipidemia, diabetes mellitus, but also related to immune-mediated inflammation [3,27]. Accumulating studies uncovered that higher levels of serum pro-inflammatory cytokines and chemokines were common in IIMs patients, which may be involved in the pathogenesis of CVD by modulating a series of mechanisms, such as induce vascular endothelial dysfunction, damage of arterial wall, vascular fibrosis and smooth muscle cell proliferation, and atherosclerotic plaque formation and rupture, eventually resulting in arterial stiffness and atherosclerosis [3,27–29]. Nevertheless, the above cardiovascular risk scores were mainly calculated based on traditional cardiovascular risk factors, which did not contain systemic inflammatory indicators. Hence, it was not surprising that the risk scores would underestimate the cardiovascular risk in IIMs patients. In this study, the positive frequency of inflammatory markers, such as ESR and CRP, was higher in patients with SCA+ than in subjects with SCA−, but there was no significant difference between the two groups. It might be attributed to the following two aspects. On the one hand, since SCA may be associated with the median of a series of measurements of these inflammatory markers, the measurement of serum ESR and CRP at a single time-point failed to identify the relationship between inflammatory markers and SCA in IIMs patients. On the other hand, the therapeutic drugs for IIMs, such as GC and DMARDs, had a certain anti-inflammatory effect, which may reduce the effect of inflammation on SCA [30].

In fact, the potential influence of GC and immunosuppressive treatment on CVD risk in IIMs remains controversial. For example, GC is the cornerstone of treatment for autoimmune diseases, but the effects of GC on cardiovascular events are often considered as a double-edged sword. Although GC could reduce inflammation and immune responses in the disease by inhibiting recruitment and migration of lymphocytes and interfering with the synthesis and secretion of pro-inflammatory cytokines, they promote the occurrence and development of traditional cardiovascular risk factors (e.g., hypertension, diabetes mellitus, and obesity) in a dose- and time-dependent manner [31,32]. In the current study, nearly half (48.8%) of IIMs patients were treated with GC, but the dosage and exposure of GC in individuals varied with the severity of the disease and the treatment regimen. This therefore made it difficult to accurately assess the relationship between GC and cardiovascular risk in IIMs patients. Some evidence pointed out that methotrexate was a potentially cardioprotective drug in rheumatic diseases, but the exact mechanism is still unclear [33]. In addition, the effect of azathioprine, cyclophosphamide, and other DMARDs has not been sufficiently confirmed. Strikingly, there was no significant difference of the use of GC and DMARDs in IIMs patients with SCA+ and SCA−, which may be attributed to the relatively small sample size in this study. Therefore, large prospective studies are required to verify the effects of GC and DMARDs in patients with an increased CV risk. Strikingly, previous studies revealed that body composition, namely lean tissue mass (LTM), fat mass, and bone mineral content, had good diagnostic values for IIMs patients, with the area under the curve (AUC) of 0.718–0.787 [34,35]. Moreover, IIMs patients had lower LTM of the upper limbs and appendicular region, higher body fat percentage, and higher android fat: gynoid fat ratio than healthy controls [35]. In addition, the altered body composition and metabolic functions in IIMs patients may be linked to increased secretion of pro-inflammatory cytokines and chemokines, such as monocyte chemoattractant protein and high sensitivity CRP [35]. Still, no link between body composition and cardiovascular risk in IIMs has been reported so far, which may provide a new direction for future research in this field.

It was worth mentioning that high-risk threshold selection may also partly explain the underestimation of carotid SCA risk in IIMs patients. As shown in Table 3, the cutoff values with best accuracy (highest Youden index) of all three risk scores were significantly lower than the preset ones, but the performance of the former was better. By applying optimal high-risk cutoff values (FRS >1.5%, SCORE >0.14%, ASCVD >1.5%), 33.7, 11.6, and 17.9% cases with SCA+ were classified as low risk. However, up to 77.9, 96.8, and 66.7% patients with SCA+ were identified as having low cardiovascular risk when the preset high risk thresholds (FRS >10%, SCORE >5%, and ASCVD >7.5%, respectively) were used. In addition, although the sensitivity of EULAR-modified risk scores (multiplied by 1.5) increased by 7.4 to 9.4%, a considerable proportion (58.3–87.4%) of patients with SCA+ were still misclassified in the low risk category. These results were also not surprising, as applying a multiplication factor of 1.5, the cutoff values of FRS, SCORE, and ASCVD were reduced to 6.7, 3.3, and 5%, and these cutoffs were significantly higher than the optimal high-risk cutoff values. Based on the above discussions, we speculate that cardiovascular risk scores at low cutoff values may be more useful in detecting IIMs patients at high cardiovascular risk, which still needs further validation.

The present study also demonstrated that carotid SCA was more prevalent in IIMs patients compared with the general population, which was in accordance with the study by Triantafyllias et al. focused on patients with antisynthetase syndrome [19]. Until now, little is known regarding the related factors of carotid SCA in patients with IIMs, but it may play a certain role in improving cardiovascular risk assessment. Therefore, we also explore the factors associated with carotid SCA in IIMs patients. The results of logistic regression analysis revealed that age was related to carotid SCA. In fact, all cardiovascular risk scores already included age. For every year increase in age, the risk of SCA in IIMs patients increases by 1.16 times (95% CI: 1.083–1.242). However, the underlying mechanism between age and carotid SCA is still unknown, which is worthy of further exploration. Similarly, studies from patients with SLE and RA pointed out that the prevalence of atherosclerotic plaques in patients was higher than in the general population, and age was associated with atherosclerotic plaques in patients with SLE (RR = 1.09, 95% CI: 1.05–1.13, *P* < 0.001) [36,37]. In addition, carotid IMT was an established risk marker for SCA and cardiovascular event, and a multicenter study reported that there was also a significant positive correlation between age and carotid IMT in antisynthetase syndrome patients (*r* = 0.697, *P* < 0.001) [19,38]. Notably, despite the results of logistic regression analysis this study revealed that there was no statistical significance among SBP, DBP, and hypertension between patients with SCA+ and those with SCA−. Vincze et al. revealed that SBP was positively correlated with carotid IMT in patients with polymyositis and dermatomyositis (*r* = 0.548, *P* = 0.006) [18]. Moreover, previous studies on other autoimmune diseases or general population had shown similar results. For example, a prospective cohort study of SLE patients also demonstrated that age and hypertension were associated with carotid SCA [39]. In addition, there was a relationship between hypertension and carotid SCA in the general population [40]. To date, the exact mechanism between hypertension and carotid SCA in patients with IIMs is unclear, which may be connected with vascular structure damage caused by arterial wall stretch with the increase of blood pressure, including vascular smooth muscle cell proliferation, vascular wall fibrosis and thickening, and increased arterial stiffness [41]. We speculate that the inconsistency between the current study and previous studies may be attributed to the relatively small sample size in this study; thus, prospective studies with large samples should be carried out to confirm the above findings.

The current study also has some limitations. First, the risk scores are originally developed in the United States or Europe; thus, the performance of these may be underestimated or overestimated in other counties. Second, the population in this study is mainly from southwest China, and the sample size of the current study is relatively small, which may not accurately represent the whole population of IIMs patients. Nonetheless, as far as we know, this is the largest study focused on cardiovascular risk assessment in IIMs patients. Third, increased carotid IMT and/or carotid plaques presence as a surrogate marker for CVD do not reflect the actual cardiovascular events. Fourth, body composition and specific serum levels of cytokines/chemokines are not included in the current study. Therefore, further prospective studies with larger sample size should be carried out to verify the results of the current study.

## 5 Conclusion

In conclusion, all cardiovascular risk scores underestimate the risk of SCA in IIMs patients, and EULAR-modified scores only provide a modest improvement in sensitivity. Age may play an important role in the development of carotid SCA among patients with IIMs. New CVD risk prediction tools of IIMs patients should be developed in the future study.
